# Study of Chinese Shadow Mapping Classification with the Application of Deep Learning Algorithms

**DOI:** 10.1155/2022/7050260

**Published:** 2022-05-25

**Authors:** Qin Fu, Qingtong Hu

**Affiliations:** ^1^Academy of Arts & Design, Department of Fine Arts, Changsha Normal University, Changsha 410100, Hunan, China; ^2^Yangtze River Cultural Heritage Institute, Hubei Institute of Fine Arts, Wuhan 430060, Hubei, China; ^3^College of Materials and Chemical Engineering, Hubei University of Technology, Wuhan 430068, Hubei, China

## Abstract

Shadow puppetry is a traditional Chinese fascinating theatre act performed by large group of artists. An artist generally uses sticks, transparent cloth screen, and flat puppets behind an illuminated background to create illusion of moving pictures during the act. These acts showcase the culture, heritage, social belief, and customs of Chinese and are a popular form of entertainment especially to youths. The modern method of digital shadow puppetry has gained a tremendous interest in the diversifying entertainment industry. Proper identification and classification of shadow puppetry is a tedious process, demanding significant research studies attention to solve the real-world vision-based problem. The proposed research studies focus on the design of artificial intelligence-based modified Grey Wolf Optimized Classifier (mGWOC) for the digital shadow puppetry problem. Data augmentation process is performed in the initial stage of the work to increase the size of the dataset used for training and testing. Secondly, to derive feature vectors from shadow puppet images, Alex Net-a deep neural network model as a part of feature extraction is adopted. Finally, Extreme Learning Classifier (ELC) is applied to allocate proper class labels. The experimental results of the proposed mGWOC reports betterment over the ResNet model, DenseNet model, and grey wolf optimization algorithm in terms of precision, recall, F-score, and kappa statistical performance measure reporting average accuracy as 0.951.

## 1. Introduction

Shadow play marks a significant place in the diversified culture of world heritage. Shadow play is a popular form of entertainment for children, adults, and elderly in many different countries. Chinese shadow puppets are the subject of this study [1]. One of the world's most well-known folk arts, Chinese shadow play is rich in cultural references. In a flat-structure shadow puppet, the joints are linked together by threads. For a moving image, a simple, lit cloth screen is used to project the shadows of puppeteers manipulating shadow puppets with sticks. Because of the need for operational skills and experience, the general public is learning less and less about China's shadow games [2]. New methods are urgently required to revitalise the Chinese shadow puppet. With the development of computerised shadow puppets, this problem can be solved. The most commonly used methods for manipulating digital shadow puppets include

1. “Controlling the puppet using a digital glove”
2. In order to manipulate the shadow puppets, computer vision is used to monitor marks on some items
3. Three-dimensional shadow puppets can be controlled directly using a multitouch interface
4. It is possible to manipulate the puppets using body movements and so forth using the Kinect sensor

Computerized shadow puppets are becoming more popular [3]. There are several ways to get the work done even if you do not have a lot of time or money. Using real-time data on human movement, people hope to develop a simple approach for creating shadow puppet animations. Pose estimation in 2D and 3D video is commonplace. To control shadow puppets, you need to know the human body's position in three dimensions. As a rigid planar component, the puppet body is modelled after a human's frontal aspect (i.e., the three quarters of the body) [4]. Shadow puppets can only move in two dimensions, but this is not the same as the two dimensions; we are used to thinking about when we talk about two-dimensional space. This is a 2.5D scene because the movement of a human body in 3D space is compressed. The 2.5D pose has one depth value for each point on the plane in a basic 3D surface representation. As a result of a possible lack of texture and depth information, recorded data on human posture cannot be used to better control shadow puppets [5]. This is not the case with a two-dimensional posture approximation, which lacks depth data. Since the movement and composition structure of human pose data for real-life scenarios and shadow play differ, it is impossible to control shadow puppets with 3D human pose data. It is possible to generalise some extraction procedures. For any depth recovery, it is important to first determine a person's position in a 2D environment. Position is estimated using two-dimensional pose techniques and deep data recovery. Using the simplified data raises the dimensionality once more. The lack of detail in gestures will have a negative impact on future mapping techniques [6]. An additional 3D human location can be approximated and then translated into a 2.5D space created by a difference in information between a real-world scene and a shadow puppet scene. A significant amount of computing power is needed to train a network to effectively analyse three-dimensional human posture. One study could incorporate both endeavours [7]. By combining 2D and 3D data on human posture, convolutional neural networks (CNNs) can be used to assess stance. As demonstrated by their own research findings, 3D human position assessment was made more accurate and faster via a structured link between body components. Traditional Chinese shadow play requires puppets to mimic all prohibited actions in order to preserve the traditional performance rhythm. Strolling and fighting are two of the most common actions of the puppets. Due to the fact that three sticks are attached to the puppet's neck and two hands, the real puppet's movements are also affected by gravity. For the past few years, some experts have been researching the use of shadow puppets in conjunction with the user's body movements [8]. However, the unique action style of a puppet is lost when using this technique. It is possible to adjust the animation by looking through a collection of shadow puppetry films and identifying and sampling different motions and actions. It is all based on the cases that have been analysed. Finally, the self-organizing network is trained in accordance with the recommendations, and the 2.5D posture data are extracted from the network as the final output [9].

For driving players in Chinese shadow dramas, 2D and 3D human posture estimation algorithms are combined. These properties are taken from 3D human pose estimation methods and mapped into 2.5D space based on comparisons between real people and shadow puppets. Going forward, there is now a modest quantity of human pose data that can be used to better drive shadow puppets. “The HOG3D feature, self-organization, and spatiotemporal consistency can all be used to improve human posture estimation networks [10].” Using the appearance of video frames, people generate temporal hint information to complete the 3D pose estimate. The 3D pose data are then constrained using the difference guide data. Among the things, we cherish most about our job which are the following:

1. This novel method to acquire 3D baseline data combines 3D human pose estimation methods with 2D human pose estimation methods.
2. Pose trajectories are translated into 2.5D space using a new translation scheme presented in this study. Before training a transformation network, people first confine the three-dimensional pose data to a 2.5-dimensional scale and then use that data as input for the transformation. In addition, certain optimization strategies aim to improve the stability, speed, and accuracy of the translation posture data when controlling shadow puppets.

Digital puppetry has been the subject of several recent investigations. During a live performance, a performer's hand gestures generate an animated character through the use of digital shadow puppetry. It takes a lot of time and effort to make a shadow puppet animation film [11]. For the first time, animators can access the body's postural data. We constructed a basic framework for digital shadow play. To interact with puppets in real time, Anim-actor uses low-cost motion capture technology based on nonexpert artists' body movements. This is how people used a semantic tagging script in Kinect to create the drive data for shadow puppets. With the use of motion planning, 2D puppets could be animated [12]. Texture mapping, mixing techniques, and blurring effects were used to animate the shadow puppets in real time. The skeleton joints were re-targeted to the shape, and a skeleton was used to drive the animation of the triangle cartoon. As long as this approach relies entirely on two-dimensional posture to move, it risks losing important motions like arm waving and horizontal body rotation. Puppet animation in 3D has just recently become a viable option. As they demonstrated, a user can simply use their own puppets to produce animated content. Using an end effector's dynamic relevance, motion capture data can be transformed into animated figures [13]. It takes both time and memory to use any of these approaches. Data on 3D human position and shadow puppet mapping are the primary subjects of this investigation. The assessment of the human stance is the most important stage in retrieving human position data [14]. A 3D human posture assessment relies mainly on photos and single parameters, methods from the past that have been employed for many years. In order to change a person's posture, they must make a series of manual changes. Stance sets that combine manual posing characteristics can be used to make inferences about a person's bodily components [15]. It is difficult to achieve typical approaches to 3D posture estimation based on a network structure. Since then, deep learning has overtaken surface learning as the go-to method. A complex self-organizing functional network is constructed through the use of deep learning architectures [16]. Pose characteristics can be used in conjunction with a variety of low-expression features to jointly estimate 3D human pose data. A single depth scan was enough to detect the 3D position of the joints in a human body. The intermediate body components are represented using an object recognition approach rather than a per-pixel approach to position estimation [17]. Reprojecting the classification result and identifying the local modes are used to create 3D projections of various body joints. Because it relies on a single image characteristic, this approach can offer inaccurate estimates when dealing with complex scenarios such as self-occlusion, mirror images, and projection distortion [18]. Three-dimensional human posture estimates can be improved by combining location data from all around the world with each body part's specific structure. Instead of relying exclusively on global position information, it was my objective to show an RGB image based on a single input of dual pose data combined with 2D and 3D pose data [19].

A deeper learning network comprising global or organised logical features has been discovered in order to improve the accuracy of 3D human posture prediction in future investigations. In order to anticipate 3D postures, researchers will need to employ 2D positions [20]. With today's advanced 2-dimensional pose estimators and methodologies, it is possible to achieve more accurate 2-dimensional poses. Among other things, a 2D pose can be used to predict a 3D pose [21]. For 3D representations, they use a nonparametric shape model to estimate 2D poses. To quickly recover 3D coordinates from 2D data, people devised a basic residual network with all nodes connected to one another.

Since this method heavily relies on 2D data from the human posture processing depth, elements of the camera's perspective may be absent. This results in an imperfect match [22]. As a result of the difficulty and effort involved in estimating a feature's exact location, the final results are rife with uncertainty. Using a technique known as many features, including global location information regression and joint detection, people can get an idea of a 3D human posture [23]. To keep the inherent consistency of space and time, 3D pose estimation is also done, utilising various features in order to keep the video 3Dmmc3 pose estimation consistent. Degrading 3D posture and building small sequences based on spatial and temporal features yield temporal information [24]. It incorporates a 3D estimation of human posture from successive video frames, which is seen above in a single image. Theobald devised a revolutionary approach to real-time attitude estimation by merging 2D and 3D data [25]. The spatial-temporal system has become more stable with the development of this method, which incorporates both global position and motion data.

## 2. Materials and Methods

Data samples collected by the courtesy of Google images are used during the classification task of digital shadow pupperty. The keywords that aided the data collection process are ‘Chinese Shadow Puppet.' Artificial intelligence-based Optimized Modified Grey Wolf Optimized Classifier (mGWOC) is the proposed study. The proposed Optimized Modified Grey Wolf Optimized Classifier (mGWOC) involves four sublayers viz data augmentation, AlexNet-based feature extraction, extreme learning machine classifier, and parameter optimization.

The detailed working of each sublayer is elaborated in the following sections.

### 2.1. Data Augmentation

Data augmentation is generally employed to increase the number of images in a dataset through the use of various transformations on the actual images. Since the models of deep learning require large training datasets, data augmentation approaches are used to improve the number of images and thereby enhance the classification accuracy. In this work, data augmentation takes place in two ways, namely, rotation and flipping.

### 2.2. Feature Extraction: The AlexNet Model

**Figure 1:**
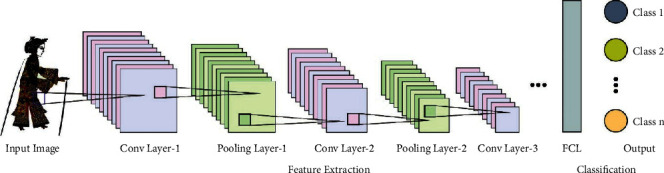
Structure of AlexNet.

During the feature extraction process, the AlexNet model is employed to derive useful feature vectors from the shadow puppet image. AlexNet is a type of convolutional neural network that contains distinct layers, namely, max pooling, input, convolution, output, and dense layers, which are its fundamental components. In 2012, it won the **Imagenet Large Scale Visual Recognition Challenge (**ILSVRC) competition [26]. It resolves the issue of image classification in which the input image is one of a thousand distinct classes and the output is a vector of class. The *k*^th^ component of the output vector is assumed to be the probability that the input image belongs to the ***k***^**th**^ class.  Figure 1 showcases the framework of AlexNet.

It is noticed that the amount of likelihoods of the whole output vector is often equivalent to one. It takes a Red Green Blue (RGB) image model as input with size 256 ∗ 256. This implicates that each image in the testing and training set must have the size of 256 ∗ 256. When the input image fails in matching the image size, it should be transformed to the normal size, that is, 256 ∗ 256 beforehand training the network. When the input image utilized is a grey-scale image, it is transformed to RGB by repeating the individual channel into a 3-channel RGB image. The structure of AlexNet is transformed from the CNN system and is utilized for computer vision-based problems. AlexNet has sixty million variables and 650,000 neurons that take a longer time to train the samples.

### 2.3. Classification: Extreme Learning Machine (ELM) Model

In this study, the ELM model receives the derived feature vectors as input and performs classification process. The training instances can be defined by

(1) $$\begin{array}{c} f\left(x\right)=\sum_{i=1}^{L}\beta_{i}h_{i}\left(x\right)=h^{T}\left(x\right)\beta , \end{array}$$

where *h*(*x*)=[*h*_1_(*x*)?*h*_*L*_(*x*)]^*T*^ indicates hidden outcome and *β* = [*β* signifies output weight.

Considering that the outcome of training instances undergoes approximation with zero error, the compact formulation can be equated in (2) as follows:

(2) $$\begin{array}{c} H\beta =t, \end{array}$$

where *H*=[*h*(*x*_1_)?*h*(*x*_*n*_)]^*T*^ denotes the hidden output matrix.

The solution of output weight comprises a linear formulation and the solution is identical to the reduction of training error, i.e., min‖.

The optimum computation of output weight can be defined by the Moore Penrose generalized inverse:

(3) $$\begin{array}{c} \hat{\beta }=H^{†}t. \end{array}$$

In general, the orthogonal projection is employed for resolving the generalized inverse, and when nonsingular, *H*^†^=*H*^*T*^(*HH*^*T*^)^−1^ is being used.

### 2.4. Parameter Tuning: Modified Grey Wolf Optimized (mGWO) Algorithm

In order to enhance the classifier results of the ELM model, the parameters such as weight and bias values are adjusted by the use of mGWO algorithm. It is utilized to increase the performance and accuracy of the grey wolf optimized method. In the study, the hunting equation and encircling equation were modified as stated in (4) and (5). The residual procedures or equations are analogous to the standard grey wolf optimized method. The primary objective of this approach is to enhance the efficacy of the motion and appropriate path of each wolf that is existing in the search region.

### 2.5. Encircling Prey

In the hunt, the prey that can be encircled by the grey wolves is improvised as follows:

(4) $$\begin{array}{c} \overset{⟶}{D}=\left|\overset{⟶}{C}\cdot \overset{⟶}{X}\left(t\right)-\theta \cdot \left(\overset{⟶}{X}\left(t\right)\right)\right|, \end{array}$$

(5) $$\begin{array}{c} \overset{⟶}{X}\left(t+1\right)={\overset{⟶}{X}}_{p}\left(t\right)-\overset{⟶}{A}\cdot \overset{⟶}{D}, \end{array}$$

where $\overset{⟶}{D}$ represent the mean, the prey location vector represented by X(t) ‘t' denotes the present iteration, and the grey wolf's location vector is represented by X(*t*+1).

$\overset{⟶}{A}$ vector and $\overset{⟶}{C}$ are represented by equations (6) and (7):

(6) $$\begin{array}{c} \overset{⟶}{A}=2\overset{⟶}{a}\cdot r_{1}-\overset{⟶}{a}, \end{array}$$

(7) $$\begin{array}{c} \overset{⟶}{C}=2\cdot {\overset{⟶}{r}}_{2}. \end{array}$$

### 2.6. Hunting

Generally, alpha and beta groups irregularly guide and participate in the hunting of prey. At first, the three optimal and best solutions of candidate are shown as in equation (8) and (9); the residual solution is represented as in equations (11), (12), and (13). All the wolf location has been improvised in the searching area by evaluating the mean of position:

(8) $$\begin{array}{c} {\overset{⟶}{D}}_{\alpha }=\left|{\overset{⟶}{C}}_{1}\cdot {\overset{⟶}{X}}_{\alpha }-\theta \cdot \overset{⟶}{X}\left(t\right)\right|, \end{array}$$

(9) $$\begin{array}{c} {\overset{⟶}{D}}_{\beta }=\left|{\overset{⟶}{C}}_{2}\cdot {\overset{⟶}{X}}_{\beta }-\theta \cdot \overset{⟶}{X}\left(t\right)\right|, \end{array}$$

(10) $$\begin{array}{c} {\overset{⟶}{D}}_{\beta }=\left|{\overset{⟶}{C}}_{3}\cdot {\overset{⟶}{X}}_{3}-\theta \cdot \overset{⟶}{X}\left(t\right)\right|, \end{array}$$

(11) $$\begin{array}{c} {\overset{⟶}{X}}_{1}={\overset{⟶}{X}}_{a}-{\overset{⟶}{A}}_{1}\cdot \left({\overset{⟶}{D}}_{\alpha }\right), \end{array}$$

(12) $$\begin{array}{c} {\overset{⟶}{X}}_{2}={\overset{⟶}{X}}_{\beta }-\overset{⟶}{A}\cdot \left({\overset{⟶}{D}}_{\beta }\right), \end{array}$$

(13) $$\begin{array}{c} {\overset{⟶}{X}}_{3}={\overset{⟶}{X}}_{\delta }{\overset{⟶}{A}}_{3}\cdot \left({\overset{⟶}{D}}_{\delta }\right), \end{array}$$

(14) $$\begin{array}{c} \overset{⟶}{X}\left(t+1\right)=\frac{{\overset{⟶}{X}}_{1}+{\overset{⟶}{X}}_{2}+{\overset{⟶}{X}}_{3}}{3}. \end{array}$$

The mGWO approach resolves a fitness factor (FF) for attaining higher classification performance. It defines the positive integer for representing the optimum efficiency of the candidate solutions. During this analysis, the minimization of the classification error rate was assumed as FF is provided in equation (15). An optimal result yields lower error rate, and the worst solution gains an improved error rate as implied by

(15) $$\begin{array}{c} \text{fitness}\left(x_{i}\right)=\text{Classifier Error Rate}\left(x_{i}\right)=\frac{\text{number of misclassified instances}}{\text{Total number of instances}}*100. \end{array}$$

## 3. Results and Discussion

**Figure 2:**
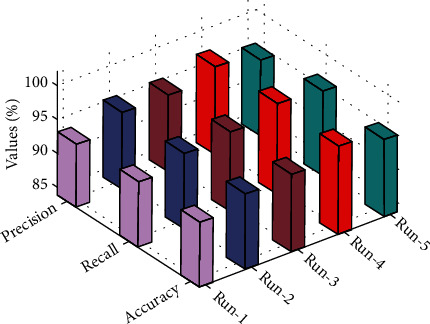
Results of statistical performance measures.

This section of the study summarizes a brief shadow puppet classification result analysis of the artificial intelligence-based modified Grey Wolf Optimized Classifier (mGWOC) technique.  Table 1 provides an overall Chinese shadow puppet classification outcome of the mGWOC technique under five distinct runs. Statistical results with respect to the performance measures precision, recall, accuracy, F-score, and kappa are tabulated. The graph represented in Figure 2 outputs the results of classification of proposed work mGWOC in terms of statistical performance measure precision, recall, and accuracy over five different simulation runs.

The results indicated that the modified Grey Wolf Optimized Classifier (mGWOC) technique has accomplished improved values of precision, recall, and accuracy. For instance, under run-1, mGWOC the technique has obtained precision, recall, and accuracy of 93.11%, 93.60%, and 93.22%, respectively. Meanwhile, under run-3, mGWOC technique has gained 94.22%, 95.68%, and 95.31%, respectively. Eventually, under run-5, mGWOC technique has accomplished 95.12%, 96.53%, and 95.31% improved performance, respectively.

**Figure 3:**
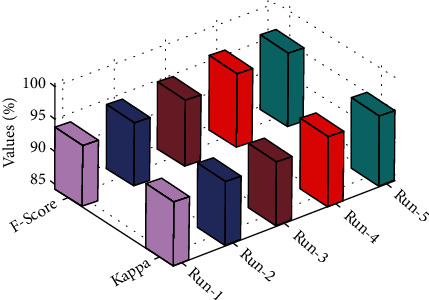
F-score and Kappa analysis of mGWOC technique.

**Figure 4:**
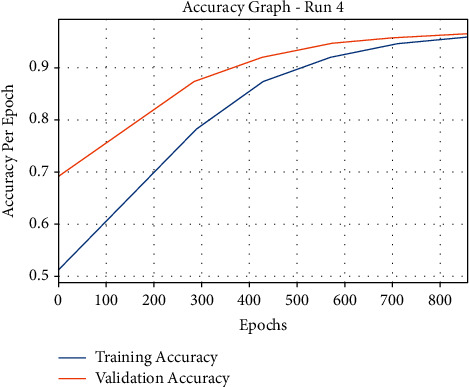
Accuracy graph analysis of AIMGWO-CSPC technique under run-4.

Figure 3 examines the classification results of the mGWOC technique in terms F-score and Kappa parameters under distinct runs. The proposed mGWOC technique resulted in 95.23% and 93.78% of F-score and Kappa, respectively. The accuracy outcome analysis of the mGWOC approach under run-4 is illustrated in Figure 4.

**Figure 5:**
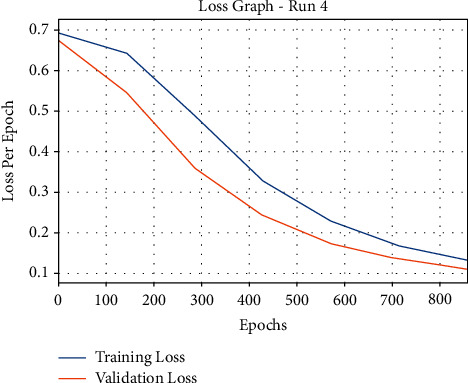
Loss graph analysis of mGWOC technique under run-4.

The results demonstrated that the mGWOC methodology has accomplished improved validation accuracy compared to training accuracy. It is also observable that the accuracy values get saturated with the count of epochs. The loss outcome analysis of the AIMGWO-CSPC technique under run-4 is shown in Figure 5. The figure exposed that the mGWOC approach has denoted the reduced validation loss over the training loss. It is additionally noticed that the loss values get saturated with the epoch count of epochs.

**Figure 6:**
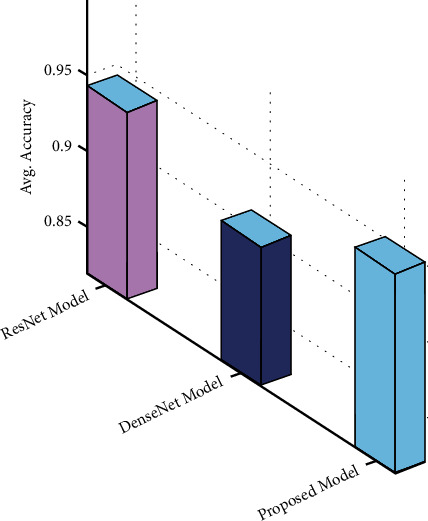
Average accuracy analysis of mGWOC technique with recent methods.

Finally, Table 2 and Figure 6 depict the average accuracy analysis of the mGWOC technique with existing ResNet and DenseNet models. The results demonstrated that the DenseNet model has accomplished lower average accuracy of 0.911. At the same time, the ResNet model has resulted in slightly increased average accuracy of 0.943.

However, the mGWOC technique has outperformed the other DL models with higher average accuracy of 0.951. By examining the abovementioned results and discussion, it is ensured that the AIMGWO-CSPC technique can attain maximum Chinese shadow puppet classification performance.

## 4. Conclusions

Chinese culture heritage association, witnessing the fading ‘shadow puppetry' folk art, increased the prominence of digital shadow puppetry in the digital era. The procedure of correctly identifying and classifying shadow puppetry is time-consuming as it is a prime research concern to present researchers in the field of artificial intelligence and wireless sensor networks. Convolutional neural network (CNN) model advancements in the last few years have made this practicable possible. This work proposes an artificial intelligence-based mean grey wolf optimization approach on the ‘Chinese Shadow Puppetry' problem. mGWOC is primarily designed to identify and classify different types of Chinese shadow puppets. This mGWOC method also uses data augmentation to begin within order to expand the dataset used for training and testing. A deep convolutional neural network model (AlexNet) is used to extract feature vectors from shadow puppet photos as a feature extraction strategy. Furthermore, modified grey wolf optimized algorithm (mGWO) with an extreme learning machine (ELM) classifier is used to assign it the appropriate class labels. Furthermore, the mGWO technique can be used to fine-tune the ELM model's weight and bias parameters. The mGWOC technique is tested on a series of test photos, and the simulation results show that the proposed technique is superior to other current approaches.

## Figures and Tables

**Table 1:** Result analysis of AIMGWO-CSPC technique with different runs.

No. of runs	Precision	Recall	Accuracy	F-score	Kappa
Run-1	93.11	93.60	93.22	93.71	94.27
Run-2	95.13	93.26	94.99	94.33	94.47
Run-3	94.22	95.68	95.31	94.56	93.65
Run-4	96.08	97.29	96.75	95.67	95.08
Run-5	95.12	96.53	95.31	95.23	93.78
**Average**	**94.73**	**95.27**	**95.12**	**94.70**	**94.25**

**Table 2:** Average accuracy analysis of AIMGWO-CSPC technique with recent methods.

Methods	Average accuracy
ResNet model	0.943
DenseNet	0.911
Proposed model	0.951

## Data Availability

The data used to support the ﬁndings of this study are available from the corresponding author upon request.
